# Relationship of family management with sociodemographic aspects and children’s physical dependence in neurological disorders^*^

**DOI:** 10.1590/1518-8345.2494.3076

**Published:** 2018-11-14

**Authors:** Gisele Weissheimer, Verônica de Azevedo Mazza, Vanessa Ferreira de Lima, Maria de Fátima Mantovani, Márcia Helena de Souza Freire, Paulo Ricardo Bittencourt Guimarães

**Affiliations:** 1 Universidade Federal do Paraná, Departamento de Enfermagem, Curitiba, PR, Brazil.; 2 Universidade Federal do Paraná, Departamento de Estatística, Curitiba, PR, Brazil.

**Keywords:** Family Nursing, Disable Persons, Child, Adolescent, Neurology, Family

## Abstract

**Objective:**

To investigate the relationship of family management with sociodemographic and physical dependence aspects of children and adolescents with neurological impairment.

**Method:**

Descriptive, cross-sectional study conducted at a child neurology center. A non-probabilistic sample was obtained from 141 family members who answered two instruments: a) Sociodemographic condition of families; b) Family Management Measure. In the statistical analysis, we used the Spearman Coefficient and the Mann Whitney Test.

**Results:**

the longer the specialized care time, the lower the identity score (rs = - 0.209, p = 0.01); the higher the effort score (rs = 0.181, p = 0.03), the family difficulty score (rs = 0.239, p = 0.001) and the impact of the disease on family life (rs = 0.213, p = 0.01). The families of children and adolescents with physical dependence for activities of daily living presented a higher score in the following dimensions: management effort (<0.001), family difficulty (p = 0.004) and perception of disease impact (p = 0.001).

**Conclusion:**

There was evidence of a correlation between management with sociodemographic and child dependence aspects, with an association between management difficulty and longer time of child and adolescent care.

## Introduction

Neurological disease in children and adolescents is a worldwide reality^1^ ^-^ ^3^. A retrospective study estimated that in the United States approximately 11% of the 960,020 hospitalizations of children aged 29 days to 19 years presented a neurological disease^4^. In Brazil, from 2002 to 2011 about 0.5% of 180,298 live births had some congenital malformation, of which 16.2% were linked to the central nervous system^5^.

In 2013, it was found that 1.3% of 200.6 million people presented physical restraint, of which 0.3% had occurred since birth and 1% was acquired. With regard to these people, 46.8% had an intense or moderate degree of limitation or could not perform normal activities of daily living^6^. Thus, it is evident that a child or adolescent with neurological impairment and physical dependence on daily tasks requires a more complex care. This calls for greater family involvement in the daily life of this population^7^.

This opens discussion on the family management, that refers to the “role of the family while responding to the disease and the various health care situations” of its member with neurological disease^8^ ^).^ Family management is anchored in the Family Management Style Framework (FMSF), with the aim of identifying how the family, as a unit of people, context and situations, responds to the illness of one of its members^9^ *.*

In 2011, an instrument originated from the FMSF, the Family Management Measure (FaMM) was published^10^. This was elaborated with the objective of complementing other forms of evaluation of family adaptation in situations of chronic diseases. This initiative also aimed to broaden its applicability to various contexts of childhood chronicity, such as type-1 diabetes^11^ ^-^ ^12^ and cancer^13^, in addition to verifying whether the family management is focused on the usual routines of life or in the demands related to the child’s disease^14^ regarding the need to change the parents’ employment for the organization of child care^15^. Such a tool facilitates the investigation of family management over time, comparing it in the different life cycles of the child and the family, thus contributing to the development of interventions in family management considered problematic^10^. Besides being a useful instrument for clinical practice, it also applicable in researches^16^, since the application of the FaMM allows knowing important aspects to be considered in the nursing professional practice^17^.

The usefulness of the FaMM has been recognized in international studies as a way to help professionals in the support to families for the management of children and adolescents in situations of chronic diseases, however, in Brazil, the researches with this instrument have been carried out for the adaptation and validation of the Family Management Measure tool^18^ ^-^ ^19^. In view of this, it would be relevant to use it to investigate family management in the context of chronic diseases in Brazil, although there are some national studies on the theme that focus mainly on the impact of diagnosis, social support and coping strategies adopted by families of children with chronic diseases^20^ ^-^ ^21^. Considering that many of these families need to change their daily lives in order to adapt to the demands generated by the child or adolescent’s disease, there must be investigation on the relationship between the sociodemographic aspects and the physical dependence of the child/adolescent with the family management.

From this perspective, the following research question was defined: Is the family management of children and adolescents with neurological impairment related to the sociodemographic and physical aspects of children and adolescents? And the following objective was drawn for this research: To investigate the relationship of family management with sociodemographic and physical dependence aspects of children and adolescents with neurological disorders.

## Method

This is a descriptive, cross-sectional study with a quantitative approach, delimited by the participation of 141 family members of children and adolescents with neurological diseases, attended at a Child Neurology Center of the Unified Health System, located in a state of southern region of Brazil. This Center is a reference at the state and provides care in several subspecialties: Autism Spectrum Disorder (ASD), Encephalopathy, Epilepsy, Recent Epilepsy, Cerebral Palsy (CP), Premature and Risk Newborn, Febrile Seizure, General Neuropediatrics, Headache and Child and Adolescent Psychiatry. To select the family members of children and adolescents, in view of the various subspecialties, we considered those with the greatest potential to respond to the objectives and inclusion criteria of this study. Thus, the following subspecialties were included: ASD, epilepsy and CP.

For the sample definition, even though this is a study with a non-probabilistic sample, we counted on the support of professionals from the Laboratory of Applied Statistics of the Federal University of Paraná. In order to do this, the months with the highest number of visits were investigated among the selected clinics in the year 2014, which preceded the planning of this study, and these values supported the definition of sample size. Thus, it was established 35 family members for ASD; 39 for epilepsy; and 67 for CP, totaling 141 relatives.

Inclusion criteria were family members of children and adolescents diagnosed or under treatment for at least six months and without hospitalizations or emergency room visits in the last two months. People who provided care and lived in the same house with the child/adolescent were considered ‘family members’, justified by daily contact and experience in care, which would allow us to identify how families incorporate the management of the child and adolescent disease in their daily routines^14^. The development of actions in hygiene, food, mobility, locomotion, elimination, health care, therapeutics, socialization and/or protection was conceived as ‘providing care’.

The criterion of the time of diagnosis, receiving care, hospital discharge or emergency room care is justified by the search for situations that are not severe^14^.

Families whose children were under the age of two and whose adolescents were above the age of 19 were excluded. This minimum age is justified for the search of situations for which the family member already had the perception of changes in growth and development of the child, while the maximum age was cut based on the definition of adolescent by the World Health Organization^22^.

Data collection was performed between May and September 2016 through face-to-face interviews, using two instruments, which were read by interviewers and answered by family members. The first instrument, named Family’s Condition, was prepared by the researchers, who had performed two pilot tests for adjustments before the beginning of the data collection. The initial pilot test was performed with six family members and the second, with five family members. It had the following variables: a) sociodemographic characterization of family members: age (years), gender and kinship to the child/adolescent; b) sociodemographic characterization of the child/adolescent: age (years) and sex; c) sociodemographic aspects of health care and social assistance: time (years) of care of the child/adolescent in the specialty of neurology, access to medications, coming from the public health system, access to the Continuous Care Grant (CCG), and access to the public transport fare exemption; e) physical dependence of the child/adolescent in the following activities of daily living (ADL): food, grooming, hygiene, bladder and bowel elimination, locomotion and mobility. In the evaluation of physical dependence in the ADL, we considered those related to the physical condition associated with the disease, and not to the phase of the child/adolescent’s development.

The second instrument used refers to the adapted and validated version of the Family Management Measure^10^, named in Brazil as “*Medida de Manejo Familiar* ^18^ ^-^ ^19^, composed of 53 items that were answered by a five-point Likert-type scale, in which one indicated ‘I totally disagree’ and five indicated ‘I totally agree’^10^. This generates a score, in which the minimum and maximum value is determined by the authors of this tool according to the number of items per dimension^23^.

The instrument is composed of 53 items in six dimensions: a) Child’s identity (five items) measures the perception of the parents about the child/adolescent and their daily life. Higher scores indicate a more normal life for the child/adolescent despite the chronic illness (minimum-maximum score: 5-25); b) Management ability (12 items) addresses the parents’ perception of their competence to take care of the child/adolescent’s illness. Higher scores mean that parents consider themselves more capable of managing the disease (minimum-maximum score: 12-60); c) Management effort (four items) addresses the work necessary to manage the condition. Higher scores indicate greater effort (minimum-maximum score: 4-20); d) Family difficulty in management (14 items) addresses the parents’ perception of the extent to which having a child/adolescent with a chronic illness makes life difficult. Higher scores indicate greater difficulty (minimum-maximum score: 14-70); e) Perception of disease impact (10 items) measures the perception of parents about the severity of the illness and its implications for the child/adolescent and the family. Higher scores indicate greater perceived severity and impact (minimum-maximum score: 10-50); f) Mutuality between parents (eight items) addresses the satisfaction of how partners/spouses work together to manage the child/adolescent’s disease. Higher scores indicate greater satisfaction (minimum-maximum score: 8-40)^10^The dimension f) is applicable exclusively to family members with partner/spouse.

In dimensions a), b) and f), higher scores indicate that the family has a more normal and easier life in managing the circumstances of the disease. And in dimensions c), d) and e), higher scores show that family life is focused on the work of managing the chronic illness of the child/adolescent and the difficulties associated with therapeutics^10^.

The data obtained from the questionnaires were organized in spreadsheets elaborated in Microsoft® Excel 2007 and double-checked. In situations where there were inconsistencies, the original questionnaire was taken up for the correction of the database.

The sociodemographic information on aspects of health care, social assistance and physical dependence were summarized by calculating absolute (n) and relative (%) frequencies; and, in the continuous variables, the mean, standard deviation (SD) and minimum and maximum values were estimated. The scores of the Family Management dimensions were calculated according to the guidelines established by the researchers who developed the instrument^23^ and presented in mean score, SD, minimum and maximum values.

To evaluate the correlation between the variables, non-parametric statistical techniques were used. The use of these techniques is justified by the lack of the normality assumption. To verify the relationship between two continuous variables (dimensions of family management versus time of specialized care in neuropediatrics), we used Spearman’s Coefficient; between a continuous and a dichotomous variable (dimensions of family management versus access to CCG, public transportation fare exemption, medications, physical dependence in activities of daily living - ADL), we used the Mann Whitney test*.*

Statistica Software version 7.0 was used in the data analysis. The results with p-value equal to or below 0.05 were considered significant (p≤0.05).

This research was approved by the Research Ethics Committee of the main institution under Opinion n. 1,299,529 and also of the co-participating institution under Opinion 1,238,692. Ethical precepts of research involving human beings were respected. All study participants were informed of the objectives of the study and signed the Informed Consent Form in two copies, guaranteeing the anonymity and privacy of the information.

## Results

Table 1 presents the sociodemographic characterization of family members and children/adolescents, aspects of health care, social assistance and physical dependence in ADL by children and adolescents with neurological disorders. The prevalent age range of family members was 31 to 40 years old, representing 48.94% (n = 69) of the interviewees. Female were 85.82% (n = 121) of the family members, and 83% (n = 117) were mothers.

**Table 1:** Sociodemographic characterization of families of children and adolescents with neurological disorders and physical dependence of children and adolescents (n = 141). Curitiba, PR, Brazil, 2016

Variable	.n (%)	Mean (SD)*	Minimum-Maximum
Age (years) of family members			
21 to 30	25 (17.73)		
31 to 40	69 (48.94)		
41 to 60	47 (33.33)	37.8 (8)	21-60
Sex of family members			
Female	121 (85.82)		
Male	20 (14.18)		
Kinship of family members with the child/adolescent			
Mother	117 (83)		
Father	19 (13)		
Others	5 (4)		
Age (years) of children/adolescents			
02 - 10	84 (60)		
11 - 17	57 (40)	9.5 (3.8)	0.5-17
Sex of children/adolescents			
Female	47 (33.00)		
Male	94 (67.00)		
Time (years) of care of children/adolescents in the specialty of neuropediatrics			
<01	9 (6.40)		
01 - 02	22 (15.60)		
03- 04	34 (24.10)		
05- 09	41 (29.10)		
10 and >	34 (24.10)		
Do not know	1 (0.70)	6.9 (4.3)	6-17
	Yes		
Access to CCG^†^	70 (49.6)		
Public access to medication	62 (44)		
Access to the public transport fare exemption	19 (13.5)		
Physical dependence of children/adolescents in activities of daily living (n=93)^‡^			
Feeding	53 (56)		
Hygiene	76 (81)		
Grooming	79 (84)		
Bladder and bowel eliminations	56 (60)		
Mobility	57 (61)		
Locomotion	59 (63)		

*SD - Standard Deviation; †CCG - Continuous Care Grant; ‡Refers only to those who were dependent on one or more activity of daily living

On the children and adolescents’ age range, 60% (n = 84) had two to 10 years and the male sex was predominant (67%; n = 94). The minimum time that the children and adolescents had been received care in neuropediatrics was six months and the maximum time was 17 years, with a mean of 6.9 years and a standard deviation of 4.3 years. In a greater proportionality, 29.1% (n = 41) of the children and adolescents had been receiving this kind of service for five and nine years. It was found that 49.6% (n = 70) of the children and adolescents received CCG and 44% (n = 62) had access to at least one medicine from the public health system.

Regarding physical dependence, 65% (n = 93) were dependent on one or more ADL. Of these, 84% (n = 79) were related to grooming and 81% (n = 76) to performing their own hygiene.

The family management scores (Table 2) were answered by the 141 family members, except for the dimension mutuality between parents, answered by 113 participants. Considering the minimum and maximum score in each dimension of this study, the management ability and mutuality between parents presented a mean score of 45.45 and 34.61, respectively, and were considered high. In the dimension family difficulty in management, there were differences between the responses of the families, indicated by the standard deviation of 12.84.

**Table 2:** Dimensions of family management of children and adolescents with neurological diseases (n=141). Curitiba, PR, Brazil, 2016

Dimension	n	Mean (SD)*	Minimum-Maximum
Child’s identity	141	15.9 (5.43)	5-25
Management ability	141	45.4 (6.24)	26-60
Management effort	141	13.2 (4.40)	4-20
Family difficulty	141	34.9 (12.84)	14-68
Mutuality between parents	113	34.6 (5.67)	9-40
Perception of disease impact	141	25.8 (6.47)	12-42

*SD - Standard Deviation

As shown in Table 3, when correlating the time of neuropediatric care of children and adolescents with the family management, there was statistical significance with the dimensions of the child’s identity, management effort, family difficulty and perception of disease impact. According to the family members’ view, the longer the time of specialized care, the lower the identity score; the greater the management effort, the family difficulty; and even more impacting is the disease in family life.

**Table 3:** Correlation of the family management with the time of care of children and adolescents in neuropediatrics (n=141). Curitiba, PR, Brazil, 2016

Dimension	.n	Rho*	p
Child’s identity	141	-0.209	0.01
Management ability	141	-0.041	0.62
Management effort	141	0.181	0.03
Family difficulty	141	0.239	0.004
Mutuality between parents	113	0.116	0.22
Perception of disease impact	141	0.213	0.01

*Rho - Spearman Correlation Coefficient

In the correlation of the family management with the physical dependence of children and adolescents in the ADL, as presented in Table 4, there was a significant relationship in the ADL of feeding, grooming and hygiene with the dimension of management ability. In the six ADLs (feeding, hygiene, grooming, bladder and bowel eliminations, mobility and locomotion), there was a significant relationship with the dimensions child’s identity, management effort, family difficulty and perception of the disease impact. The families of the dependent children/adolescents in the ADL presented a higher mean score of management effort, family difficulty and perception of the disease impact. The families of independent individuals in the ADL presented a higher mean score in the dimension of child’s identity and management ability.

**Table 4:** Relationship of family management with physical dependence of children and adolescents in activities of daily living (n = 141). Curitiba, PR, Brazil, 2016

Dimension	C*	Feeding				Grooming				Hygiene				Bladder and bowel eliminations				Mobility				Locomotion		
		n	Mean (SD)^†^	p^‡^		n	Mean (SD)^†^	p^‡^		.n	Mean (SD)^†^	p^‡^		n	Mean (SD)^†^	p^‡^		n	Mean (SD)^†^	p^‡^		n	Mean (SD)^†^	p^‡^
Child’s identity	I^§^	88	17,6 (5,44)	<0.001		62	18,4 (5,45)	<0.001		65	18,4 (5,23)	<0.001		85	17,8 (5,18)	<0.001		84	17,7 (5,26)	<0.001		82	17,8 (5,29)	<0.001
	D^¶^	53	13,2 (4,18)			79	14,0 (4,55)			76	13,8 (4,67)			56	13,1 (4,52)			57	13,3 (4,52)			59	13,3 (4,48)	
Management ability	I^§^	88	46,5 (6,28)	0,001		62	47,1 (5,84)	0,003		65	47,1 (6,04)	0,003		85	46,2 (6,2)	0,05		84	46,2 (6,02)	0,05		82	46,2 (6,13)	0,06
	D^¶^	53	43,5 (5,76)			79	44,1 (6,26)			76	44,0 (6,09)			56	44,2 (6,17)			57	44,3 (6,44)			59	44,3 (6,28)	
Management effort	I^§^	88	11,8 (4,45)	<0.001		62	10,7 (4,27)	<0.001		65	11,1 (4,37)	<0.001		85	11,9 (4,4)	<0.001		84	12,0 (4,48)	<0.001		82	12,0 (4,52)	<0.001
	D^¶^	53	15,5 (3,20)			79	15,2 (3,41)			76	15,0 (3,54)			56	15,2 (3,61)			57	15,0 (3,62)			59	14,9 (3,67)	
Family difficulty	I^§^	88	32,1 (12,9)	<0.001		62	30,1 (12,1)	<0.001		65	30,4 (12,7)	<0.001		85	31,9 (12,4)	<0.001		84	32,5 (12,6)	0,004		82	32 (12,6)	<0.001
	D^¶^	53	39,6 (11,2)			79	38,7(12,11)			76	38,8 (11,7)			56	39,5 (12,1)			57	38,4 (12,3)			59	38,9 (12,0)	
Mutuality between parents	I^§^	71	35,3 (5,49)	0,08		48	35,2 (4,59)	0,76		52	35,2 (5,54)	0,37		67	35,0 (5,49)	0,45		67	35,1 (5,52)	0,28		67	35,1 (5,52)	0,28
	D^¶^	42	33,4 (5,84)			65	34,1 (6,35)			61	34,0 (5,76)			46	33,9 (5,93)			46	33,8 (5,85)			46	33,8 (5,85)	
Perception of disease impact	I^§^	88	23,7 (6,39)	<0.001		62	23,0 (6,5)	<0.001		65	22,8 (6,42)	<0.001		85	23,8 (6,14)	<0.001		84	23,7 (6,23)	<0.001		82	23,7 (6,3)	<0.001
	D^¶^	53	29,2 (5,03)			79	28,0 (5,56)			76	28,3 (5,36)			56	28,8 (5,80)			57	28,8 (5,54)			59	28,6 (5,63)	

*C - Condition; †SD - Standard Deviation; ‡ p - Mann Whitney test; § I- Independent ¶ D - Dependent

Table 5 shows the correlation between the dimensions of family management with the access or non-access to governmental, social and welfare subsidies by children and adolescents with neurological diseases. There was a significant statistical correlation between CCG and the dimensions of management ability, management effort, family difficulty and perception of the disease impact. In this sense, the families that did not receive the CCG presented higher mean score of management ability. On the other hand, those who received it showed higher mean scores of management effort, family difficulty and perception of the disease impact.

**Tabela 5:** Relação do manejo familiar com o acesso aos subsídios governamentais sociais e assistenciais por crianças e adolescentes com doenças neurológicas (n=141). Curitiba, PR, Brasil, 2016

Dimension	Condition	CCG*				Transportation Fare Exemption				Medicine		
		.n	Mean (SD)^†^	p^‡^		.n	Mean (SD)^†^	p^‡^		.n	Mean (SD)^†^	p^‡^
Child’s identity	Receive	71	16,7 (5,69)	0,11		122	15,9 (5,37)	0,75		79	16,9 (5,39)	0,02
	Do not receive	70	15,1 (5,06)			19	15,8 (5,91)			62	14,7 (5,27)	
Management ability	Receive	71	46,7 (5,9)	0,01		122	45,8 (6,09)	0,02		79	46,3 (6,14)	0,05
	Do not receive	70	44,1 (6,34)			19	42,6 (6,67)			62	44,2 (6,21)	
Management effort	Receive	71	12,3 (4,47)	0,01		122	13,0 (4,5)	0,13		79	12,3 (4,48)	0,003
	Do not receive	70	14,2 (4,15)			19	14,7 (3,45)			62	14,4 (4,04)	
Family difficulty	Receive	71	32,0 (12,51)	0,004		122	34,5 (12,98)	0,31		79	33,4 (13,52)	0,071
	Do not receive	70	37,9 (12,56)			19	37,5 (11,88)			62	36,8 (11,75)	
Mutuality between parents	Receive	62	34,7 (5,69)	0,82		100	34,5 (5,65)	0,5		65	34,9 (4,6)	0,83
	Do not receive	51	34,4 (5,7)			13	35,2 (6,03)			48	34,1 (6,88)	
Perception of disease impact	Receive	71	24,1 (6,22)	<0.001		122	25,7 (6,72)	0,7		79	25,2 (6,67)	0,16
	Do not receive	70	27,5 (6,31)			19	26,2 (4,63)			62	26,5 (6,17)	

*CCG - Continuous Care Grant; †SD - Standard Deviation; ‡p - Mann Whitney test

In families where the children/adolescents did not receive a public transportation fare exemption, there was a statistically significant relationship with the management ability dimension, in which the mean score was higher than those who received it. The families of the children/adolescents who received at least one medicine were significantly correlated with the dimensions child’s identity and management effort. Those who did not receive medication had a higher mean score in the child’s identity dimension.

## Discussion

Researches have consistently found that children and adolescents with neurological disorders require various forms of care that generate challenges for their family members to manage them on a daily basis^24^ ^-^ ^25^. In this research, we investigated the relationship of family management with sociodemographic aspects of health care, social assistance and physical dependence among children and adolescents.

The sociodemographic conditions of health care, social assistance of the families and the situation of physical dependence/independence of children and adolescents in ADL were significantly associated with all dimensions of family management, except in the dimension of mutuality between parents.

In this study, the child’s identity score was negatively correlated with the highest time of scare in a specialized neurology service, indicating a change in the perception of the family in relation to the child/adolescent and to the daily life of the family. During child and adolescent development, as the years pass, families start to perceive the intensity of the development deficit^26^. So, the family’s expectation that individuals perform activities in an autonomous way may not be met, since this public will always need the help of their relatives to carry them out due to their limitations related to the disease^27^.

The child’s identity was positively correlated with the physical independence of children/adolescents in ADL and lack of support for receiving medication from the public health system. Many children/adolescents use monotherapy, others polytherapy, situations that are linked to the most severe clinical condition^28^. Those who do not use medications may have minor impairments, thus interfering directly with the child/adolescent’s perception of identity.

The management ability score was positively associated with the families when their children did not receive the CCG, did not obtain exemption of transportation fare and were independent for the ADL of grooming and feeding. Therefore, these family members felt more capable to perform the necessary care^10^. The aforementioned benefits are linked to the low-income population^29^ ^-^ ^30^; thus, it is understood that the greater purchasing power allows access to resources to support the care for children and adolescents.

With regard to the social grant of transportation exemption for the families that need it, it is verified that having availability and accessibility to transportation contributes to improve families’ lives, since it allows the displacement for multiprofessional consultation and for the social activities outside the domestic scope^31^. However, in this research, it was found that families with higher management ability scores did not use this benefit. This benefit has a direct correlation with family income^30^; in view of this, the families that do not receive the benefit have greater purchasing power. Family members perceived themselves more capable of caring for those who did not present physical limitations for feeding, hygiene and grooming activities. Children and adolescents’ demands may require the reduction or interruption of work activities of family members, with consequent decrease in family income^32^, making families need social support.

Fine motor activities, such as self-care, involving hygiene and grooming, are difficult areas for individuals with neurological impairments. Also, more severe neurological conditions affect more strongly children’s bimanual ability^33^.

The family difficulty score indicates parents’ perception of how having a child with illness makes life more difficult^10^. This was negatively correlated with longer time of care in a specialized service of neurology and with those families in which the children/adolescents presented dependence in the ADL and, in a positive way, with the families that did not receive the CCG.

Pediatric involvement due to the symptoms of the disease causes the caregiver to spend long periods meeting the needs of children and, thus, to move away from social interaction, family and friends^34^. In addition, family members incessantly seek general and specialized treatment, sometimes facing challenges in order to achieve them due to the limited number of such procedures^35^ ^)^ and unavailability of public services^36^.

The functional limitations of locomotion, when associated with lower socioeconomic conditions, can generate greater financial loss due to higher costs with assistance technologies, for locomotion, home adaptations, transportation and instructions/trainings of family members to use these technologies^37^, making families face intense difficulties. It was verified in a study that a large part of the families of children/adolescents with greater functional dependence received CCG^38^.

The family effort score refers to the perception of the work they develop to manage the disease^10^. This was negatively correlated with a longer time of service in specialized neurology service, the receiving of CCG and of medication from the public health system.

This result is in line with another study carried out in China with 538 caregivers of children/adolescents at more advanced ages, in which there was greater difficulty in family management^39^. It is common for children and adolescents with neurological disease to require continuous specialized care due to their motor, visual, hearing, neurological, psychiatric and neurosurgical follow-up and drug therapy^40^. Thus, the family routine encompasses the mobility for services of physiotherapy, equine therapy, hydrotherapy, occupational therapy, speech therapy, among others^32^, which requires from the family greater efforts to meet all the demands of care. In addition, individuals with motor deficit often need assistance through a wheelchair, walker and even the support of another person^40^, so that the greater the physical dependence, the more intense are the activities of care for the family members^41^.

In a study of 610 children who used wheelchairs in the home environment, 88% (n = 537) of them were manual, 2% (n = 11) had electric chair and 10% (n = 62) were mixed. Of the 599 children who used a manual chair, 28% (n = 165) of the children and adolescents were able to move by themselves and 72% (n = 434) needed a person to push them^42^. Mothers with older and dependent children state that care is more laborious due to the development of actions such as bathing, assisting in the use of the toilet, grooming and moving the child^43^.

The children and adolescent population with complex medical conditions, such as neurological ones, makes continuous use of medicines^44^, which requires attention from the family to administer them, as well as to obtaining them. The exhausting routine causes many family members to stop working because it is not possible to combine an occupational activity and the care of the child^45^.

Another variable correlated as greater effort for families was the CCG, in Brazil, as a social assistance policy that supports families in basic needs and which, in many situations, is their only source of income^46^. The economic condition of the families is related to the maintenance of the children and adolescents’ therapy^37^.

The score of the perception of disease impact indicates the family’s perception of the severity of the disease and its complications regarding the health of the children/adolescents and the families. This dimension was negatively correlated with the longer time of service in specialized neurology service, the receiving of CCG and with the situations in which the children/adolescents were dependent for the ADL. Higher scores indicate greater severity and family’s concern in managing the disease.

The future implications of the neurological condition commonly involve the parents’ concern about the future life of these individuals, on who will take care of them in their absence^28^, on aspects related to professional life, in which the performance of these children and adolescents is related to the evolution of the disease^47^. In this aspect, the intellectual quotient is a predictive factor for socialization and professionalization; however, motor independence may be related to the ability to maintain a job^48^. The implications of transition to independent adult life involve the housing condition^49^ and married life, which, because of illness, may lead parents to think that children will be rejected and unable to maintain a marriage relationship^50^.

In addition, there is concern about money management for the maintenance of treatment of children and adolescents with neurological diseases^51^. In the present study, families receiving CCBs presented greater concern and perceived the disease as more impacting. This factor may be related to the situation of lower income and to the disease. These findings are consistent with previous research that indicates that sociodemographic aspects of families and children/adolescents influence family management^15^ ^,^ ^39^ ^,^ ^52^.

The mean scores of the dimensions of family management obtained in this study were compared with those provided by other surveys of families in other situations of chronic diseases. In an investigation with families of children/adolescents surviving cancer^13^ and another focusing on diabetes mellitus^11^, the scores of child’s identity and management ability were higher when compared to the families of the present study. On the other hand, the dimensions of management effort and perception of disease impact in the study of diabetes mellitus had a mean of 13.82 and 25.55, respectively^11^, and family difficulty in the study of children surviving cancer had a mean score of 34.60^13^, which were similar to those of this study. The management effort score of children/adolescents surviving cancer was 10.01^13^, thus, lower than that of this survey.

In addition, as in this study, others presented greater standard deviation in the family difficulty dimension than in the other dimensions^13^ ^,^ ^15^. In view of the dissimilarity between the responses of the families regarding management difficulties, we suggest further qualitative research that seeks to understand these difficulties.

The present research showed aspects that facilitated and made difficult the family management, which affect the adaptation of the families to the demands of care of the child and the adolescent with neurological disease. The recognition of these aspects by professionals makes it possible to use knowledge and skills relevant to this area of knowledge for the improvement of child/adolescent health and to help families to adapt to conditions resulting from child/adolescent disease.

This study had some limitations, such as the cross-sectional nature of data collection that does not allow evaluating cause and effect between variables; the non-probabilistic sample; and despite the attempt to include different family members, the respondents were mainly mothers, so it was not possible to analyze family management from the perspective of other members.

## Conclusion

There was a correlation between family management and sociodemographic and physical dependence aspects. This association showed that the management ability of the families was positive in the conditions where the children/adolescents did not need social grants, access to medications from the public health system and were physically independent for the ADL. There was also an association between the difficulty of management and the longer time of care for children and adolescents. This issue implies the need for families to be cared for beyond the period of impact of the diagnosis and the acute phase. For this purpose, nursing needs to rethink the practice that meets the health needs of this phase, including a continuous care proposal, providing families with support in this change of life and health condition, which occurs over time in the chronic disease. This can lead to a change in their capacity to manage such phenomena.

The use of the FaMM has the potential to provide information on social and care needs of children/adolescents as families begin to manage the disease situation. This tool can help nursing professionals to identify how families deal with the disease and, thus, to propose strategies to deal, in a more adequate way, with the relatives in the advance of a more effective management of the child/adolescent with a chronic disease.
